# More than Traits? Time Perspective, Academic Achievement, and Educational Expectations Among Adolescents

**DOI:** 10.3390/bs16081310

**Published:** 2026-08-01

**Authors:** Ilke Bayazitli, Zena R. Mello, Shasta M. Ihorn, Frank C. Worrell

**Affiliations:** 1 Berkeley School of Education, University of California, Berkeley, CA 94720-1670, USA; frankc@berkeley.edu; 2 Psychology Department, College of Science & Engineering, San Francisco State University, San Francisco, CA 94132, USA; zmello@sfsu.edu (Z.R.M.); sihorn@sfsu.edu (S.M.I.)

**Keywords:** time perspective, academic achievement, educational expectations, personality traits, adolescents

## Abstract

In the current study, we examined the associations between four time perspective constructs (time attitudes, time frequency, time relation, and time orientation) and two academic outcomes (self-reported academic achievement and educational expectations), controlling for the Big Five personality traits and demographics (age, gender, and maternal education). Participants included 758 adolescents (*M*_age_ = 15.81, *SD*_age_ = 1.22) from the Western United States. Several subscales had associations with academic achievement and educational expectations above and beyond personality. Specifically, negative attitudes toward the future were negatively associated with academic achievement, and positive attitudes toward the future were positively associated with educational expectations. Present negative attitudes were also significantly associated with academic achievement, though this association was not practically meaningful. The remaining time attitude subscales, time frequency, time relation, and time orientation did not survive Bonferroni correction. Overall, the effect sizes were limited for most of the identified statistically significant associations, suggesting minimal practical significance. These findings identify specific time perspective dimensions that may warrant further longitudinal and intervention research to evaluate their potential role in promoting academic outcomes among adolescents.

## 1. Introduction

Adolescence has been described as “an age of opportunity” because this stage of the lifespan includes remarkable malleability (Steinberg, 2014). Academic achievement is a primary domain of adolescent development as it has been associated with lifelong success and well-being (Johnson et al., 2011). For example, academic achievement in adolescents is correlated with subjective well-being in adulthood (Olsson et al., 2013), and educational expectations in adolescence are associated with higher educational and occupational attainment in adulthood (Jacob & Wilder, 2010). Given the significance of academic outcomes for adolescents, it is important to identify psychosocial factors that support academic outcomes. Some scholars have suggested that time perspective may be such a construct (Mello, 2019; Pawlak & Moustafa, 2023).

Although some researchers have found associations between academic variables and time perspective constructs (e.g., Andretta & Worrell, 2022; Prow et al., 2016), some scholars have argued that time perspective is an aspect of personality traits (Gjesme, 1983; Strathman et al., 1994). Thus, it is important to determine if time perspective’s associations with other constructs are subsumed under personality, or if time perspective has unique associations which will support its feasibility as a unique intervention target in its own right (Mello, 2019; Worrell et al., 2021). As such, in the current study, we examined the associations of multiple time perspective dimensions with academic achievement and educational expectations in an adolescent sample, controlling for demographic variables and personality traits. Before introducing our specific hypotheses, we first provide a brief overview of two contemporary conceptualizations of time perspective and the literature on the association of time perspective constructs with academic outcomes. Next, we discuss personality constructs and their associations with time perspective. We aim to clarify the associations between these two constructs with the current study.

### 1.1. Conceptualizations of Time Perspective

The time perspective literature has several definitions and measures of time perspective, which has resulted in concerns about the comparability of findings (Kooij et al., 2018). For instance, a substantial number of early studies focused on future time perspective and other future-oriented constructs (Kooij et al., 2018; Lens et al., 2012; Pawlak & Moustafa, 2023). The focus on constructs such as optimism (Scheier & Carver, 1985), hope (Adelabu, 2008; Snyder et al., 1996), and future orientation (Cottle, 1969; Seginer, 1992) resulted in a more restricted conceptualization of time perspective. In response, Zimbardo and Boyd (1999) adopted Lewin’s (1951, p. 75) conceptualization of time perspective as “the totality of the individual’s view of his psychological future and psychological past existing at a given time.” They suggested that solely focusing on one time period, such as the future, does not assess the breadth of the time perspective construct (Zimbardo & Boyd, 1999) and developed the Zimbardo Time Perspective Inventory (ZTPI). Although the ZTPI is one of the most frequently used instruments in the time perspective literature, numerous concerns have been raised about the robustness of ZTPI scores (e.g., McKay et al., 2020; Worrell & Mello, 2007; Worrell et al., 2018), leading to the development of other theoretical formulations. Also drawing from Lewin’s (1939, 1951) definition, Mello and Worrell (2015) put forward a conceptual model that underscored the multidimensional nature of time perspective and developed the Adolescent and Adult Time Inventory (AATI; Mello & Worrell, 2007) to operationalize the five subconstructs in their model: time attitudes, time frequency, time relation, time orientation, and time meaning. *Time attitudes* are positive and negative feelings about the three time periods (Worrell et al., 2013). *Time frequency* refers to the frequency of thoughts about the time periods (Mello et al., 2009). *Time relation* refers to the perceived relationship among the time periods, and *time orientation* focuses on the relative importance individuals place on the three time periods (Mello et al., 2013). Finally, time meaning refers to how individuals define time (Mello et al., 2009). In this study, we used the Mello and Worrell (2015) conceptualization of time perspective, drawing upon four of the constructs assessed with the AATI: that is, time attitudes, which focus on feelings, and time frequency, time orientation, and time relation, which focus on thoughts.

### 1.2. Time Perspective and Academic Outcomes

There are several studies in which researchers examined the association between temporal constructs and academic achievement. For example, Hong (2025) found that future time perspective had a direct relationship with academic achievement as well as a mediated relationship via increased academic engagement and reduced academic burnout. Other future-oriented constructs, such as future orientation (Brown & Jones, 2004; Seginer & Mahajna, 2004) and hope (Adelabu, 2008; Dixson, 2019) have also been found to be positively related to academic outcomes.

Researchers have also examined the associations between *feelings* about time and academic achievement. For example, students with a positive time attitude profile reported higher grade point averages (GPA) than those with other profiles in a sample of female adolescents from New Zealand (Alansari et al., 2013). Alansari et al. (2013) findings were replicated in two studies with adolescent samples from the United States, in which meaningful associations were found between positive time attitude profiles and GPA (Andretta et al., 2014; Prow et al., 2016). Importantly, Prow et al. (2016) used achievement data from school records, whereas other studies have relied on student-reported achievement. In a more recent study, Andretta and Worrell (2022) found that academically talented students with different time attitudes did not differ in the amount and perception of difficulty of work that they had in an intense summer program, but students in the positive profile did report enjoying the program more and expected to obtain higher grades. Several studies have shown that time attitudes are also associated with academic outcomes other than achievement among adolescents. For instance, Andretta et al. (2014) found meaningful associations between positive time attitude profiles and educational expectations. Positive time attitude profiles were also associated with higher academic self-concept and school belonging (Worrell & Andretta, 2019).

To our knowledge, only two studies that used the AATI have examined the associations between *thoughts* about time and academic achievement. Mello et al. (2009) found that past time frequency was positively associated with academic achievement, with more frequent thoughts on the past being associated with higher GPA. A balanced time orientation (i.e., considering the past, the present, and the future as equally important) was also related to a higher GPA (Mello et al., 2013). By highlighting multiple time periods, these findings supplement the extant literature, which has only reported on the association between achievement outcomes and thoughts about the future (see Pawlak & Moustafa, 2023 for a review). Additionally, the association between time relation and academic achievement has received limited attention. In the current study, we investigated the associations between multiple time perspective dimensions and academic achievement and educational expectations.

### 1.3. Time Perspective and Personality Traits

The conceptual association between time perspective and personality traits has sparked debates in the literature. For example, some scholars have contended that personality subsumes time perspective (Rudzinska-Wojciechowska et al., 2021). Others have defined time perspective as a static dispositional trait comparable to personality (Gjesme, 1983; Strathman et al., 1994). In contrast, other scholars have argued that time perspective is a dynamic and malleable construct that is distinct from personality (Carstensen, 2006; Seijts, 1998). And a fourth perspective describes time perspective as a multifaceted construct that comprises both static and dynamic characteristics (Stolarski et al., 2014; Zimbardo & Boyd, 1999).

The associations between time perspective, as measured by the ZTPI, and personality traits have been examined in several empirical studies (Adams & Nettle, 2009). For example, using the ZTPI, positive associations were found between (a) future time perspective and conscientiousness, (b) present fatalistic and past negative time perspectives and neuroticism, and (c) past positive time perspective and extraversion and agreeableness (Kairys, 2010; Zimbardo & Boyd, 1999). However, because the correlations were modest to moderate (Daugherty & Brase, 2010), time perspective was posited to be a distinct construct from personality (Kairys & Liniauskaite, 2015).

Indeed, results from a number of studies have supported the distinctiveness of time perspective from personality. For example, in samples of college students, ZTPI scores have been found to be associated with life satisfaction (Zhang & Howell, 2011) and holding positive judgments about the self and life (Przepiorka et al., 2020), controlling for personality traits. Similarly, AATI time attitude scores were associated with substance use in adolescents above and beyond personality traits (Assylkhan et al., 2021). In the Assylkhan et al. (2021) study, correlations between AATI time attitude scores and the Big Five personality traits ranged from |0.05| to |0.52| (*Mdn* = |0.21|), with correlations being stronger between emotional stability and time attitudes (*Mdn* = |0.25|) than the other personality traits (*Mdn* ≤ |0.21|).

To date, time perspective and personality have been examined in relation to achievement in only one study. Rudzinska-Wojciechowska et al. (2021) predicted exam performance and GPA with ZTPI scores, after controlling for gender, fluid and verbal intelligence, and the Big Five personality traits. Time perspective scores added 7% to the prediction of exam performance and 3% to the prediction of GPA, with Present Fatalistic having a meaningful coefficient in the first equation and Future Positive in the second equation. If intelligence had not been controlled, it is possible that time perspective variables may have accounted for more variance.

### 1.4. The Present Study

In this study, we posed the following research question: Does time perspective account for unique variance in academic outcomes, after controlling for demographic variables and personality traits? We expected that time perspective would be associated with academic outcomes above and beyond personality traits, given prior research including both on time perspective and personality traits (Assylkhan et al., 2021; Rudzinska-Wojciechowska et al., 2021). We hypothesized that positive time attitudes would be positively associated with academic outcomes and negative time attitudes would have an inverse relationship (e.g., Andretta et al., 2014). For time frequency, we hypothesized that more frequent thoughts about the future would be positively associated with academic outcomes (Pawlak & Moustafa, 2023). For time relation, we hypothesized that perceiving temporal periods as linearly related or interrelated to one another would be positively associated with academic outcomes (Mello et al., 2013). For time orientation, we hypothesized that a greater emphasis on the present and future or an equal emphasis on all temporal periods would be associated with higher academic outcomes (Mello et al., 2013).

## 2. Method

### 2.1. Participants

The initial sample consisted of 791 participants, with the final sample excluding 33 participants over the age of 18 (*M* = 15.81; *SD* = 1.22, range = 12–18). The majority of the participants identified as female (56%), followed by male (42%) and non-binary or transgender (2%). Racial/ethnic groups included the following: African American/Black (6%), American Indian/Alaskan Native (<1%), Asian American/Pacific Islander (17%), European American/White (14%), Hispanic/Latinx (42%), multiethnic (15%), and other racial/ethnic groups (5%). The average maternal education of the sample, which was used as a proxy for socioeconomic status, was between a high school diploma and an Associate’s Degree (*M* = 2.58, *SD* = 1.16) on a scale that ranged from no high school diploma/GED to a doctoral degree.

### 2.2. Procedure

The research team recruited participants by visiting classes in two public high schools in the Western United States and giving speeches about the study. Interested students were provided with materials that included a parental consent and participant assent forms and a survey. Participants returned the study materials to the research team at the high school. Anonymity was ensured by providing separate envelopes for the completed surveys. Data were collected in 2015 and 2016. Each participant was compensated with $10. The response rate was 68%. The surveys were completed on the adolescents’ own time. The study was approved by the Institutional Review Board (IRB) of San Francisco State University (IRB #: H15-33c). The study was not preregistered, so the findings should be treated as exploratory.

### 2.3. Measures

#### 2.3.1. Time Perspective

The AATI (Mello & Worrell, 2007) was used to assess multiple time perspective dimensions including time attitudes, time frequency, time relation, and time orientation.

##### Time Attitudes

The time attitude subscales of the AATI-TA (Mello & Worrell, 2007) were used to measure time attitudes. The AATI-TA consists of six 5-item subscales that assess positive and negative feelings about the past, present, and future: Past Positive (e.g., “My past is full of happy memories”), Past Negative (e.g., “My past makes me sad”), Present Positive (e.g., “I am pleased with the present”), Present Negative (e.g., “I am not satisfied with my present”), Future Positive (e.g., “My future makes me smile”), and Future Negative (e.g., “Thinking about my future makes me sad”). Participants respond on a 5-point Likert scale ranging from 1 (*totally disagree*) to 5 (*totally agree*), with higher mean scores indicating more positive or negative feelings about the temporal period. Past research has provided evidence of reliability and validity of AATI-TA scores (e.g., Moon et al., 2023; Worrell et al., 2013), and scores were also reliable in this sample (see Table 1).

##### Time Frequency

Time frequency was measured with three single-item variables on the AATI (Mello & Worrell, 2007) using a 5-point Likert scale ranging from 1 (*almost never*) to 5 (*almost always*). Participants were asked to indicate how often they thought about the past (past frequency), present (present frequency), and future (future frequency).

##### Time Relation

Time Relation (AATI-TR; Mello & Worrell, 2007) was measured with a single item with four response options. Each response option included a figure that represented the past, present, and future as circles, with overlapping circles representing an association. Participants were asked to choose one figure that represented how they understand the time periods to be related. The figures display the following time relations: Unrelated (all the time periods are unrelated), Present-Future (the present and the future are related), Linear (the past is related to the present, and the present is related to the future), and Interrelated (all of the time periods are related to one another).

##### Time Orientation

Time Orientation (AATI-TO; Mello & Worrell, 2007) consisted of a single item with seven response options. Each response option depicted the time periods as circles, with larger circles indicating more importance and smaller circles indicating less importance. Participants were asked to choose the option that reflects how relatively important they perceived the time periods. Figures represented the following time orientation types: Past Oriented, Present Oriented, Future Oriented, Past and Future Oriented, Past and Present Oriented, Present and Future Oriented, and Balanced (i.e., all three time periods are important).

#### 2.3.2. Academic Outcomes

Two academic outcomes were measured: academic achievement and educational expectations.

##### Academic Achievement

Academic achievement was measured with two items. The first item asked the participants to report their GPA on a 4-point, continuous scale. The second item asked, “Generally, what grades do you get in your classes?” Response options ranged from 1 (*A’s*) to 5 (*F’s*) and were reverse coded in the analyses so that higher values would reflect higher achievement. 2 A composite score of academic achievement was generated by calculating the average of the standardized scores of students’ self-reported GPA and letter grades. Prior research has shown that adolescents can report accurate information about their academic achievement (Crockett et al., 1987).

##### Educational Expectations

Educational expectations were measured with one item. The question asked, “How much schooling do you expect to complete?” Response options ranged from 1 (*high school diploma*) to 6 (*doctorate*).

#### 2.3.3. Personality Traits

Personality traits were measured with the Adolescent Personality Style Inventory (APSI; Lounsbury et al., 2003). The APSI consists of 48 items across five subscales: Agreeableness (10 items; e.g., “I am always polite to other people”), Conscientiousness (9 items; e.g., “I like to plan things before I do them”), Emotional Stability (9 items; e.g., “I sometimes feel like I’m going crazy”), Extraversion (9 items; e.g., “I smile a lot when I am around other people”), and Openness (11 items; e.g., “I like to visit new places”). Participants responded to each item on the APSI using a 5-point Likert scale ranging from 1 (*strongly disagree*) to 5 (*strongly agree*). Fifteen items are reverse coded. The subscale scores are the average of the items that comprise the subscale. Lounsbury et al. (2003) reported internal consistency estimates >0.80 for the five APSI scores. Specifically, scores on all subscales of the measure have yielded good to excellent internal consistency (αs > 0.80). In the current study, internal consistency estimates indicated that the scores on the personality subscales had alpha values >0.75, with the exception of the Agreeableness subscale (α = 0.55). In keeping with previous research (Assylkhan et al., 2021), four items on the Agreeableness subscale were removed. The internal consistency estimate for scores on the revised Agreeableness subscale was 0.74.

## 3. Results

### 3.1. Preliminary Analyses

Skewness and kurtosis levels were within the acceptable range for all continuous variables (see Table 1). As shown in Table 2, the most common time relation responses were interrelated, present-future related, and linearly related. As shown in Table 3, the most common time orientation responses were present-future, balanced, and past-future.

Regarding missingness, time perspective dimensions (i.e., time attitudes, time frequency, time relation, and time orientation) were analyzed for missing data, and some missingness was observed. Missingness was at or below approximately 10% across all subscales of time perspective, with the future negative subscale showing the highest rate (10.16%). Subscale scores were computed as the mean of available items, so participants with partial item-level responses were retained in the analyses. A case was excluded from a regression model only when the composite score for the relevant time perspective dimension was entirely missing. Participants with at least one missing response reported lower academic achievement, *t*(738) = 4.57, *p* < 0.001, *d* = 0.35 than their counterparts. Participants with missing responses did not differ from the rest of the sample in their educational expectations, *t*(704) = 1.51, *p* = 0.13, *d* = 0.12.

### 3.2. Primary Analyses

One-way analyses of variance were conducted to examine how individuals with different views of time relation and time orientation differed on academic achievement and educational expectations. Results in Table 2 and Table 3 indicate statistically significant but not practically meaningful differences among the time relation and time orientation groups.

Regression analyses were used to examine the association between time constructs and the two academic outcomes. To account for Type I error, a Bonferroni correction was applied at the omnibus level at α = 0.0125 (0.05/4). For dimensions whose omnibus Δ*R*^2^ survived this threshold, individual subscale coefficients were examined and evaluated using effect sizes. Standardized regression coefficients with absolute values of 0.20 or larger were considered to reflect practically meaningful effects (Ferguson, 2009). Step 1 of each model included the Big Five personality traits along with three demographic covariates (age, gender, and maternal education). Step 1 was significant for both outcomes and is reported in full in Table S1. Notably, maternal education was a practically meaningful predictor of academic achievement across all models (B values ≥ 0.23), and gender was statistically significant, with female participants showing higher achievement and educational expectations, but not meaningful (βs ≤ 0.15). Age was not meaningfully associated with the educational variables across models. Variance inflation factors for all predictors in the regression models ranged from 1.06 to 3.27, indicating that multicollinearity was not a concern.

Table 4 and Table 5 show the results of the hierarchical regression analyses for the associations among time attitudes, academic achievement, and educational expectations, controlling for demographic variables and personality traits. Separate analyses were done for each of the four time perspective dimensions (i.e., time attitudes, frequency, relation, and orientation), with the subscales simultaneously entered in the models. Regarding academic achievement, Step 2 indicated that future negative time attitude scores were meaningfully associated with academic achievement, controlling for demographic variables and personality traits. Maternal education and conscientiousness were also practically meaningful predictors. Regarding educational expectations, future positive time attitude scores had the highest coefficient after maternal education, but the coefficient did not meet the threshold for practical meaningfulness.

Table 6 and Table 7 show the results of the hierarchical regression analyses for the associations of time frequency to academic achievement and educational expectations, controlling for demographic variables and personality traits. The omnibus tests for time frequency were not statistically significant for either outcome, and no time frequency variable was meaningfully associated with academic achievement or educational expectations. Both conscientiousness and maternal education were practically meaningful predictors of academic achievement, and maternal education was the only practically meaningful predictor of educational expectations.

Table 8 and Table 9 show the results of the hierarchical regression analyses for the associations of time relation choices to academic achievement and educational expectations, controlling for demographic variables and personality traits. The interrelated time relation option was used as the reference category based on prior research (Mello et al., 2013). The results indicated that time relation was not significantly associated with either outcome above and beyond demographic variables and personality traits. As in the previous analyses, conscientiousness and maternal education predicted academic achievement and maternal education predicted educational expectations.

Table 10 and Table 11 show the results of the hierarchical regression analyses for the associations of time orientation choices to academic achievement and educational expectations, controlling for demographic variables and personality traits. The balanced time orientation option was used as the reference category based on prior research (Mello et al., 2013). Results showed that time orientation was not significantly associated with academic achievement or educational expectations, with the same pattern of associations for maternal education and conscientiousness seen in the previous analyses.

## 4. Discussion

The salience of academic outcomes in adolescence for subsequent attainment and well-being has led researchers to investigate factors that promote academic outcomes. Time perspective has been identified as a potential mechanism to foster academic outcomes (Mello, 2019; Zimbardo & Boyd, 1999). To address the potential utility of time perspective in promoting academic outcomes, we examined the associations of several time perspective constructs with academic achievement and educational expectations, controlling for demographic variables and personality traits. The results of the hierarchical regression analyses indicated that some time perspective dimensions were associated with academic achievement and educational expectations above and beyond personality traits, though the practical significance of the effects varied across dimensions and outcomes.

Regarding time attitudes, future negative time attitudes showed a practically meaningful negative association with academic achievement (β = −0.25), though this coefficient was attenuated compared to models without demographic covariates (β = −0.35). The coefficient for present negative attitudes also approached practical meaningfulness, but effects were small for the other four time attitudes. For educational expectations, future positive time attitudes had the highest *non-meaningful* coefficient of the time attitude and personality variables. These modest effect sizes suggest that time attitudes may be more strongly associated with academic outcomes when treated as profiles rather than as individual subscales. This distinction matters given that time attitudes are conceptually interrelated and complementary. Accordingly, a variable-centered approach that isolates each subscale’s unique association may not fully illustrate how these dimensions are related to academic outcomes when considered together. In some prior research (e.g., Alansari et al., 2013; Worrell & Andretta, 2019), practically meaningful associations were not observed between time attitude scores and academic outcomes, although time attitude profiles did show practically meaningful associations.

Time frequency was not meaningfully associated with academic achievement and educational expectations. These findings suggest that this aspect of time perspective conceptualized by Mello and Worrell (2015) differs from other conceptualizations of future time perspective and research to establish time frequency correlates needs to be conducted. Several researchers (e.g., Adelabu, 2008; Janeiro et al., 2017) have reported associations between future time perspective and academic achievement. However, Adelabu (2008) did not control for personality constructs or demographic variables, and the association between future time perspective and achievement in Adelabu’s study was statistically significant but non-meaningful (*r* = 0.12). Janeiro et al. controlled for gender only, and the effect of time perspective on achievement was mediated by approaches to learning. Given present and future frequency’s associations with future time attitudes, examining a mediated relationship should be an avenue for future investigations.

Neither time relation nor time orientation was meaningfully associated with academic achievement or educational expectations, controlling for demographic variables and personality. However, research on both of these constructs is quite limited to date. The limited research available has revealed interesting patterns. Adolescents who saw the time periods as interrelated reported significantly lower risky behavior (e.g., getting trouble with the police) scores than those who saw the time periods as not connected (Mello et al., 2017), although the effect size was modest (*g* = 0.31). Similarly, adolescents who saw the three time periods as important (i.e., a balanced view; *g* = 0.31) or who thought that the present and future were most important (*g* = 0.42) reported engaging in less risky behavior than those who also focused on the present. Thus, these constructs may be associated with adaptive behavioral functioning, even if they are not associated with academic outcomes. Additional research is needed to clarify the associations between time relation and time orientation and outcomes among adolescents.

Overall, the current findings provide limited support for the unique contribution of some time perspective dimensions, specifically time attitudes, to academic outcomes above and beyond demographic variables and personality traits, broadly consistent with prior work on the separability of time perspective from personality (Assylkhan et al., 2021; Przepiorka et al., 2020). However, no support was found for a practically meaningful association between educational outcomes and the other three time perspective dimensions—that is time frequency, time relation, and time orientation. This pattern of dimension-specific associations suggests that academic outcomes are likely not the product of any single cognitive, dispositional, or temporal factor, but instead emerge from a more complexly determined set of interacting variables. As such, time attitudes may warrant further investigation as potential intervention targets in future longitudinal or experimental research. Additional research is needed to examine the role of other time perspective dimensions, as well as demographic variables, particularly using profile-based approaches that can more fully capture the complexity of how individuals feel and think about time.

### Limitations and Future Directions

The current study includes some limitations. The data are cross-sectional, which limits the knowledge about the directionality of the findings. Future studies should employ a longitudinal research design to observe changes over time. Furthermore, the sample includes only two schools from the same geographic region. Further research could include more representative samples across the United States as well as other countries, thanks to the worldwide availability of the time perspective measures used in the current study. Future studies could also include multidimensional profile analyses to better capture how individuals feel and think about time in association with academic outcomes. Also, several central variables were measured with single items, including time frequency subscales, time relation, time orientation, and educational expectations, which may have introduced measurement error and may attenuate observed associations. Similarly, as several time orientation categories had small numbers, results should also be interpreted considering the limited power. As the data in the current study were collected in 2015–2016, although the theoretical framework and measures remain current, future studies should replicate these findings with more recent samples. Missing data bias also remains important given that participants with higher missingness did report lower academic achievement. Finally, further studies may examine the utility of interventions that focus on specific time perspective dimensions to support academic outcomes. Given that most effects were small, the current evidence supports a limited and dimension-specific contribution of time perspective. As time attitudes showed the most consistent associations with academic outcomes, future research might examine whether targeting negative future time attitudes could support academic achievement, while treating the remaining time perspective dimensions as warranting further longitudinal examination before being pursued as intervention targets.

## Figures and Tables

**Table 1:** Descriptive Statistics and Internal Consistency Estimates for Time Perspective, Academic Outcomes, and Personality Traits.

	*N*	*M*	*SD*	Minimum	Maximum	Skewness	Kurtosis	*α*
Time attitudes								
Past positive	752	3.38	0.80	1.00	5.00	–0.35	2.92	0.83
Past negative	752	2.75	0.90	1.00	5.00	0.28	2.55	0.85
Present positive	753	3.42	0.76	1.00	5.00	–0.35	2.95	0.85
Present negative	753	2.69	0.83	1.00	5.00	0.32	2.91	0.85
Future positive	753	3.70	0.82	1.00	5.00	–0.45	3.02	0.88
Future negative	754	2.38	0.85	1.00	5.00	0.43	2.92	0.83
Time frequency								
Past frequency ^a^	750	3.47	0.95	1.00	5.00	–0.45	3.30	—
Present frequency ^a^	752	3.75	1.00	1.00	5.00	–0.68	3.19	—
Future frequency ^a^	751	4.06	0.96	1.00	5.00	–0.97	3.63	—
Academic outcomes								
Academic achievement ^a^	740	–0.04	0.97	–3.87	1.65	–0.80	3.41	—
Educational expectations ^a^	706	4.49	1.30	1.00	6.00	–0.99	3.62	—
Personality traits								
Agreeableness	728	3.57	0.69	1.00	5.00	–0.47	3.18	0.74
Conscientiousness	727	3.33	0.70	1.00	5.00	–0.29	3.69	0.86
Emotional stability	728	2.99	0.73	1.11	5.00	–0.03	2.97	0.83
Extraversion	728	3.26	0.64	1.00	5.00	–0.05	3.07	0.76
Openness	728	3.48	0.71	1.00	5.00	–0.68	4.18	0.87

^a^ Single-item variables.

**Table 2:** Time Relation and Academic Outcomes.

Time Relation		Distribution ^a^	Academic Achievement	Educational Expectations
		*n* (%)	*M* (*SD*)	*M* (*SD*)
1. Unrelated	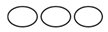	69 (10)	–0.24 (0.87)	4.21 (1.32)
2. Present-future related	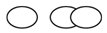	207 (30)	–0.08 (1.01)	4.37 (1.40)
3. Linearly related	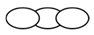	188 (27)	0.04 (0.93)	4.66 (1.20)
4. Interrelated	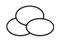	235 (34)	0.13 (0.91)	4.62 (1.15)
*F* ratio			3.57 *	3.35 *
η^2^			0.02 [0.00, 0.04] ^b^	0.02 [0.00, 0.03] ^b^

^a^ The percentages do not add up to 100 due to rounding. ^b^ 95% confidence interval. * *p* < 0.05.

**Table 3:** Time Orientation and Academic Outcomes.

Time Orientation		Distribution	Academic Achievement	Educational Expectations
		*n* (%)	*M* (*SD*)	*M* (*SD*)
1. Past	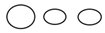	17 (2)	0.03 (0.78)	4.81 (1.05)
2. Present	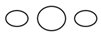	49 (7)	–0.11 (0.82)	4.41 (1.26)
3. Future	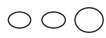	83 (12)	–0.11 (0.96)	4.39 (1.37)
4. Past-future	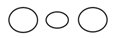	102 (15)	–0.13 (1.10)	4.55 (1.24)
5. Past-present	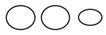	22 (3)	0.00 (0.82)	4.30 (1.30)
6. Present-future	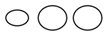	303 (44)	0.13 (0.90)	4.54 (1.29)
7. Balanced	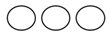	120 (17)	–0.04 (1.00)	4.55 (1.25)
*F* ratio			1.58	0.45
η^2^			0.01 [0.00, 0.03] ^a^	0.00 [0.00, 0.01] ^a^

^a^ 95% confidence interval.

**Table 4:** Associations Between Time Attitudes and Academic Achievement, Controlling for Demographic Variables and Personality Traits.

Variable	Academic Achievement						
	*b*	*SE*	95% CI		β	*R*^2^adj	Δ*R*^2^
			*LL*	*UL*			
Time attitudes						**0.277**	**0.046**
Past positive	–0.03	0.06	–0.14	0.09	–0.02		
Past negative	–0.11	0.05	–0.22	–0.00	–0.10		
Present positive	0.16	0.07	0.02	0.30	0.13		
Present negative	0.20	0.07	0.07	0.33	0.18		
Future positive	–0.03	0.06	–0.15	0.08	–0.03		
Future negative	–0.28	0.06	–0.40	–0.16	**–0.25**		
Demographic variables							
Age	–0.02	0.03	–0.07	0.04	–0.02		
Gender	0.19	0.05	0.09	0.29	0.13		
Maternal education	0.16	0.02	0.12	0.20	**0.28**		
Personality traits							
Agreeableness	0.04	0.07	–0.10	0.17	0.03		
Conscientiousness	0.27	0.06	0.15	0.39	**0.20**		
Emotional stability	–0.03	0.06	–0.14	0.09	–0.02		
Extraversion	–0.22	0.06	–0.34	–0.10	–0.15		
Openness	0.15	0.07	0.02	0.29	0.11		
Intercept					—		

Note. Step 2 of the hierarchical regression analyses is shown. *N* = 666. Step 1 included Big Five personality traits, age, gender, and maternal education and is reported in Table S1. Bolded values indicate practically meaningful coefficients (Ferguson, 2009).

**Table 5:** Associations Between Time Attitudes and Educational Expectations, Controlling for Demographic Variables and Personality Traits.

Variable	Educational Expectations						
	*b*	*SE*	95% CI		β	*R*^2^adj	Δ*R*^2^
			*LL*	*UL*			
Time attitudes						**0.179**	**0.043**
Past positive	0.00	0.09	–0.17	0.17	0.00		
Past negative	0.11	0.08	–0.05	0.28	0.08		
Present positive	–0.08	0.11	–0.28	0.13	–0.04		
Present negative	0.06	0.10	–0.13	0.26	0.04		
Future positive	0.25	0.09	0.07	0.42	0.16		
Future negative	–0.20	0.09	–0.37	–0.02	–0.13		
Demographic variables							
Age	–0.10	0.04	–0.18	–0.02	–0.09		
Gender	0.26	0.08	0.11	0.40	0.13		
Maternal education	0.20	0.03	0.14	0.26	**0.24**		
Personality traits							
Agreeableness	0.14	0.10	–0.05	0.34	0.07		
Conscientiousness	0.05	0.09	–0.13	0.23	0.03		
Emotional stability	0.07	0.09	–0.10	0.23	0.04		
Extraversion	–0.20	0.09	–0.38	–0.03	–0.10		
Openness	0.19	0.10	–0.01	0.40	0.10		
Intercept					—		

Note. Step 2 of the hierarchical regression analyses is shown. *N* = 651. Step 1 included Big Five personality traits, age, gender, and maternal education and is reported in Table S1. Bolded values indicate practically meaningful coefficients (Ferguson, 2009).

**Table 6:** Associations Between Time Frequency and Academic Achievement, Controlling for Demographic Variables and Personality Traits.

Variable	Academic Achievement						
	*B*	*SE*	95% CI		β	*R*^2^adj	Δ*R*^2^
			*LL*	*UL*			
Time frequency						**0.231**	0.007
Past frequency	0.04	0.04	–0.04	0.11	0.04		
Present frequency	0.07	0.04	–0.00	0.14	0.07		
Future frequency	–0.02	0.04	–0.09	0.06	–0.02		
Demographic variables							
Age	–0.02	0.03	–0.07	0.04	–0.02		
Gender	0.18	0.05	0.08	0.28	0.13		
Maternal education	0.18	0.02	0.14	0.22	**0.31**		
Personality traits							
Agreeableness	0.08	0.07	–0.06	0.21	0.06		
Conscientiousness	0.27	0.06	0.14	0.39	**0.20**		
Emotional stability	0.10	0.05	0.01	0.20	0.08		
Extraversion	–0.17	0.06	–0.30	–0.05	–0.12		
Openness	0.18	0.07	0.04	0.33	0.14		
Intercept					—		

Note. Step 2 of the hierarchical regression analyses is shown. *N* = 657. Step 1 included Big Five personality traits, age, gender, and maternal education and is reported in Table S1. Bolded values indicate practically meaningful coefficients (Ferguson, 2009).

**Table 7:** Associations Between Time Frequency and Educational Expectations, Controlling for Demographic Variables and Personality Traits.

Variable	Educational Expectations						
	*B*	*SE*	95% CI		β	*R*^2^adj	Δ*R*^2^
			*LL*	*UL*			
Time frequency						**0.152**	0.014
Past frequency	0.04	0.06	–0.07	0.15	0.03		
Present frequency	0.06	0.05	–0.04	0.17	0.05		
Future frequency	0.12	0.05	0.01	0.23	0.09		
Demographic variables							
Age	–0.09	0.04	–0.17	–0.02	–0.09		
Gender	0.28	0.08	0.13	0.43	0.14		
Maternal education	0.19	0.03	0.13	0.25	**0.23**		
Personality traits							
Agreeableness	0.12	0.10	–0.08	0.32	0.06		
Conscientiousness	0.08	0.09	–0.10	0.25	0.04		
Emotional stability	0.12	0.07	–0.02	0.26	0.07		
Extraversion	–0.15	0.09	–0.32	0.03	–0.07		
Openness	0.23	0.11	0.02	0.44	0.12		
Intercept					—		

Note. Step 2 of the hierarchical regression analyses is shown. *N* = 643. Step 1 included Big Five personality traits, age, gender, and maternal education and is reported in Table S1. Bolded values indicate practically meaningful coefficients (Ferguson, 2009).

**Table 8:** Associations Between Time Relation and Academic Achievement, Controlling for Demographic Variables and Personality Traits.

Variable	Academic Achievement						
	*B*	*SE*	95% CI		β	*R*^2^adj	Δ*R*^2^
			*LL*	*UL*			
Time relation						**0.244**	0.012
Unrelated	–0.28	0.12	–0.51	–0.04	–0.09		
Present-future related	–0.23	0.08	–0.39	–0.07	–0.12		
Linearly related	–0.15	0.08	–0.32	0.01	–0.07		
Interrelated	Reference category						
Demographic variables							
Age	–0.01	0.03	–0.07	0.04	–0.02		
Gender	0.16	0.05	0.06	0.26	0.11		
Maternal education	0.19	0.02	0.14	0.23	**0.32**		
Personality traits							
Agreeableness	0.04	0.07	–0.09	0.18	0.03		
Conscientiousness	0.31	0.06	0.19	0.43	**0.23**		
Emotional stability	0.09	0.05	–0.00	0.18	0.07		
Extraversion	–0.15	0.06	–0.27	–0.03	–0.10		
Openness	0.21	0.07	0.07	0.35	0.15		
Intercept	–2.08	0.53	–3.12	–1.05	—		

Note. Step 2 of the hierarchical regression analyses is shown. *N* = 621. Step 1 included Big Five personality traits, age, gender, and maternal education and is reported in Table S1. Bolded values indicate practically meaningful coefficients (Ferguson, 2009).

**Table 9:** Associations Between Time Relation and Educational Expectations, Controlling for Demographic Variables and Personality Traits.

Variable	Educational Expectations						
	*b*	*SE*	95% CI		β	*R*^2^adj	Δ*R*^2^
			*LL*	*UL*			
Time relation						**0.160**	0.008
Unrelated	–0.24	0.17	–0.58	0.10	–0.06		
Present-future related	–0.15	0.12	–0.38	0.09	–0.05		
Linearly related	0.11	0.12	–0.13	0.34	0.04		
Interrelated	Reference category						
Demographic variables							
Age	–0.09	0.04	–0.16	–0.01	–0.08		
Gender	0.25	0.07	0.10	0.39	0.13		
Maternal education	0.20	0.03	0.14	0.26	**0.25**		
Personality traits							
Agreeableness	0.08	0.10	–0.11	0.28	0.04		
Conscientiousness	0.13	0.09	–0.05	0.30	0.07		
Emotional stability	0.04	0.07	–0.09	0.18	0.02		
Extraversion	–0.12	0.09	–0.29	0.05	–0.06		
Openness	0.34	0.10	0.14	0.55	0.17		
Intercept	3.32	0.76	1.82	4.82	—		

Note. Step 2 of the hierarchical regression analyses is shown. *N* = 611. Step 1 included Big Five personality traits, age, gender, and maternal education and is reported in Table S1. Bolded values indicate practically meaningful coefficients (Ferguson, 2009).

**Table 10:** Associations Between Time Orientation and Academic Achievement, Controlling for Demographic Variables and Personality Traits.

Variable	Academic Achievement						
	*b*	*SE*	95% CI		β	*R*^2^adj	Δ*R*^2^
			*LL*	*UL*			
Time orientation						**0.239**	0.007
Past	0.09	0.23	–0.36	0.53	0.01		
Present	–0.07	0.15	–0.37	0.22	–0.02		
Future	–0.05	0.12	–0.30	0.19	–0.02		
Past-future	–0.09	0.12	–0.32	0.14	–0.04		
Past-present	0.07	0.20	–0.32	0.46	0.01		
Present-future	0.10	0.09	–0.09	0.29	0.05		
Balanced	Reference category						
Demographic variables							
Age	–0.02	0.03	–0.07	0.04	–0.02		
Gender	0.17	0.05	0.07	0.27	0.12		
Maternal education	0.19	0.02	0.14	0.23	**0.33**		
Personality traits							
Agreeableness	0.06	0.07	–0.08	0.19	0.04		
Conscientiousness	0.28	0.06	0.16	0.40	**0.21**		
Emotional stability	0.07	0.05	–0.02	0.17	0.06		
Extraversion	–0.17	0.06	–0.29	–0.06	–0.12		
Openness	0.22	0.07	0.08	0.36	0.16		
Intercept	–2.07	0.53	–3.12	–1.02	—		

Note. Step 2 of the hierarchical regression analyses is shown. *N* = 619. Step 1 included Big Five personality traits, age, gender, and maternal education and is reported in Table S1. Bolded values indicate practically meaningful coefficients (Ferguson, 2009).

**Table 11:** Associations Between Time Orientation and Educational Expectations, Controlling for Demographic Variables and Personality Traits.

Variable	Educational Expectations						
	*b*	*SE*	95% CI		β	*R*^2^adj	Δ*R*^2^
			*LL*	*UL*			
Time orientation						**0.154**	0.003
Past	0.15	0.33	–0.50	0.79	0.02		
Present	–0.12	0.22	–0.54	0.31	–0.02		
Future	–0.16	0.18	–0.52	0.20	–0.04		
Past-future	0.04	0.17	–0.29	0.37	0.01		
Past-present	0.02	0.29	–0.55	0.60	0.00		
Present-future	–0.00	0.14	–0.27	0.26	–0.00		
Balanced	Reference category						
Demographic variables							
Age	–0.09	0.04	–0.17	–0.02	–0.09		
Gender	0.26	0.08	0.11	0.41	0.14		
Maternal education	0.20	0.03	0.14	0.26	**0.25**		
Personality traits							
Agreeableness	0.11	0.10	–0.09	0.31	0.06		
Conscientiousness	0.11	0.09	–0.07	0.28	0.06		
Emotional stability	0.06	0.07	–0.08	0.20	0.03		
Extraversion	–0.13	0.09	–0.30	0.05	–0.06		
Openness	0.35	0.11	0.14	0.55	0.18		
Intercept	3.30	0.78	1.78	4.82	—		

Note. Step 2 of the hierarchical regression analyses is shown. *N* = 609. Step 1 included Big Five personality traits, age, gender, and maternal education and is reported in Table S1. Bolded values indicate practically meaningful coefficients (Ferguson, 2009).

## Data Availability

The data that support the findings of this study are available upon request from Zena R. Mello. The data are not publicly available due to the privacy of research participants.

## Supplementary Materials

The following supporting information can be downloaded at: https://www.mdpi.com/article/10.3390/bs16081310/s1, Table S1: Step 1 Hierarchical Regression Results, Table S2: Bonferroni-Corrected Correlations Among Study Variables.
