# Supplementary material for: More than Traits? Time Perspective, Academic Achievement, and Educational Expectations Among Adolescents
Source: Behav Sci (Basel). 2026 Aug 1;16(8):1310. doi: 10.3390/bs16081310 (PMC13509214; doi:10.3390/bs16081310)
Supplement: Supplementary file 1 [file behavsci-16-01310-s001.zip › behavsci-4355516-supplementary.pdf]

Supplementary Materials

Table S1

Step 1 Hierarchical Regression Results

Variables	*B*	SE	β	*t*	*p*	95% CI
**Academic Achievement** (Adjusted R^2 = .234)						
Agreeableness	.088	.068	.064	1.29	.198	[–.046, .221]
Conscientiousness	.268	.063	**.202**	4.28	<.001	[.145, .392]
Emotional Stability	.091	.047	.070	1.93	.054	[–.002, .184]
Extraversion	–.187	.061	–.128	–3.09	.002	[–.306, –.069]
Openness	.207	.071	.155	2.91	.004	[.068, .347]
Age	–.012	.027	–.015	–0.44	.663	[–.065, .041]
Gender	.182	.051	.128	3.58	<.001	[.082, .282]
Maternal Education	.189	.021	**.322**	9.19	<.001	[.148, .230]
**Educational Expectations** (Adjusted R^2 = .144)						
Agreeableness	.142	.100	.075	1.42	.156	[–.054, .338]
Conscientiousness	.073	.092	.039	0.79	.428	[–.107, .253]
Emotional Stability	.088	.069	.050	1.28	.200	[–.047, .224]
Extraversion	–.156	.088	–.077	–1.76	.079	[–.329, .018]
Openness	.275	.105	.147	2.63	.009	[.069, .482]
Age	–.094	.039	–.088	–2.38	.018	[–.171, –.016]
Gender	.295	.075	.149	3.91	<.001	[.148, .442]
Maternal Education	.200	.030	**.250**	6.67	<.001	[.141, .260]

Note. Standardized coefficients ≥ |0.20| are bolded.

Table S2

Bonferroni-Corrected Correlations Among Study Variables

Variable	1	2	3	4	5	6	7	8	9	10	11	12	13	14	15	16
1. Age	—															
2. Maternal education	–.02	—														
3. Past frequency	.11	.09	—													
4. Present frequency	.05	.17	**.30**	—												
5. Future frequency	.08	.05	**.27**	**.33**	—											
6. Past positive	–.03	.11	–.02	.16	.13	—										
7. Past negative	.05	–.10	**.20**	–.07	.00	**–.55**	—									
8. Present positive	.03	.07	–.10	**.22**	.10	**.48**	**–.32**	—								
9. Present negative	.03	.00	**.27**	–.05	.04	**–.26**	**.53**	**–.63**	—							
10. Future positive	.04	.04	.04	**.20**	**.32**	**.31**	–.08	**.56**	**–.33**	—						
11. Future negative	.02	–.15	.07	–.16	–.16	–.13	**.42**	**–.34**	**.58**	**–.53**	—					
12. Agreeableness	.10	.13	.16	**.23**	**.24**	.18	–.14	**.21**	–.17	**.25**	**–.29**	—				
13. Conscientiousness	.01	–.01	.11	.18	.19	.16	–.05	.18	–.13	**.25**	–.19	**.62**	—			
14. Emotional stability	–.03	.00	**–.30**	–.06	–.09	**.24**	**–.42**	**.40**	**–.52**	.17	**–.32**	–.10	**–.21**	—		
15. Extraversion	.04	.04	.04	.12	.15	**.23**	–.17	**.26**	**–.20**	**.30**	**–.26**	**.46**	**.35***	–.00	—	
16. Openness	.04	.17	.18	**.24**	**.25**	.19	–.07	**.20**	–.09	**.30**	**–.23**	**.65**	**.62**	**–.26**	**.51**	—

Note. Given the sample size, all correlations ≥ |.12| are significant at the .001 level. Coefficients ≥ |0.20| are bolded.
